# Perpendicular implantation of porcine trachea extracellular matrix for enhanced xenogeneic scaffold surface epithelialization in a canine model

**DOI:** 10.3389/fsurg.2022.1089403

**Published:** 2023-01-12

**Authors:** Ayumu Kato, Tetsuhiko Go, Yasuhiro Otsuki, Naoya Yokota, Chang Sung Soo, Noriyuki Misaki, Toshiki Yajima, Hiroyasu Yokomise

**Affiliations:** Department of General Thoracic, Breast and Endocrine Surgery, Faculty of Medicine, Kagawa University, Kagawa, Japan

**Keywords:** airway defect repair, biomaterial, extracellular matrix, porcine trachea, tracheal regeneration

## Abstract

**Objective:**

The availability of clinically applied medical materials in thoracic surgery remains insufficient, especially materials for treating tracheal defects. Herein, the potential of porcine extracellular matrix (P-ECM) as a new airway reconstruction material was explored by xenotransplanting it into a canine trachea.

**Methods:**

P-ECM was first transplanted into the buttocks of Narc Beagle dogs (*n* = 3) and its overall immuno-induced effects were evaluated. Subsequently, nine dogs underwent surgery to create a tracheal defect that was 1 × 2 cm. In group A, the P-ECM was implanted parallel to the tracheal axis (*n* = 3), whereas in group B the P-ECM was implanted perpendicular to the tracheal axis (*n* = 6). The grafts were periodically observed by bronchoscopy and evaluated postoperatively at 1 and 3 months through macroscopic and microscopic examinations. Immunosuppressants were not administered. Statistical evaluation was performed for Bronchoscopic stenosis rate, graft epithelialization rate, shrinkage rate and ECM live-implantation rate.

**Results:**

No sign of P-ECM rejection was observed after its implantation in the buttocks. Bronchoscopic findings showed no improvement concerning stenosis in group A until 3 months after surgery; epithelialization of the graft site was not evident, and the ECM site appeared scarred and faded. In contrast, stenosis gradually improved in group B, with continuous epithelium within the host tissues and P-ECM. Histologically, the graft site contracted longitudinally and no epithelialization was observed in group A, whereas full epithelialization was observed on the P-ECM in group B. No sign of cartilage regeneration was confirmed in both groups. No statistically significant differences were found in bronchoscopic stenosis rate, shrinkage rate and ECM live-implantation rate, but graft epithelialization rate showed a statistically significant difference (G-A; sporadic (25%) 3, vs. G-B; full covered (100%) 3; *p* = 0.047).

**Conclusions:**

P-ECM can support full re-epithelialization without chondrocyte regeneration, with perpendicular implantation facilitating epithelialization of the ECM. Our results showed that our decellularized tracheal matrix holds clinical potential as a biological xenogeneic material for airway defect repair.

## 1. Introduction

Airway defects or fistula affecting the trachea and/or bronchi that occur during or after respiratory surgery are difficult to treat and may cause life-threatening conditions (1). Artificial tracheae and tracheal regeneration to compensate for tracheal/bronchial defects have been studied for 70 years since they were first reported in 1948 (2). Since then, various methods of producing artificial trachea and promoting tracheal regeneration have been tested in both large and small animals (3); however, to date, none have been widely applied clinically (4, 5). Various artificial and biological airway materials have been proposed for airway defect repair, with varying degrees of success. Nonetheless, artificial materials have various problems, such as absorption and incorporation into the body. Additionally, usage of biomaterials includes allogeneic transplantation, which is problematic in terms of supply when applied to humans (6, 7). Hence, an ideal material to repair tracheal injuries and defects is still warranted in routine practice.

Conconi et al. first reported the potentials of tracheal matrices produced using a detergent-enzymatic method as useful scaffolds for tissue engineering (8). Further experiments revealed the unique characteristics of these matrices, such as their flexibility and histocompatibility, and no antigenicity even on the back of mice of heterologous animals (9). Therefore, in the present study, we used a porcine extracellular matrix (P-ECM) to repair tracheal defects and considered its application as a xenogeneic biomaterial. Although various studies have explored the potential of ECM as a xenogeneic material (10), there are few reports on large animals. To expand our knowledge on the potential of P-ECM for clinical practice, we selected dogs as the experimental model. Furthermore, because P-ECM is nonantigenic, it can be used as a graft without the need for immunosuppression after surgery.

## 2. Materials and methods

Twelve female Narc Beagle dogs, weighing 9.25 ± 0.25 kg, underwent surgery. All dogs were purchased from the Kitayama Shizuoka Laboratory Animal Center (Shizuoka, Japan). Porcine tracheae for P-ECM preparation were purchased from Tokyo Shibaura Zouki Corp. (Tokyo, Japan).

### 2.1. Study design

**Figure 1:**
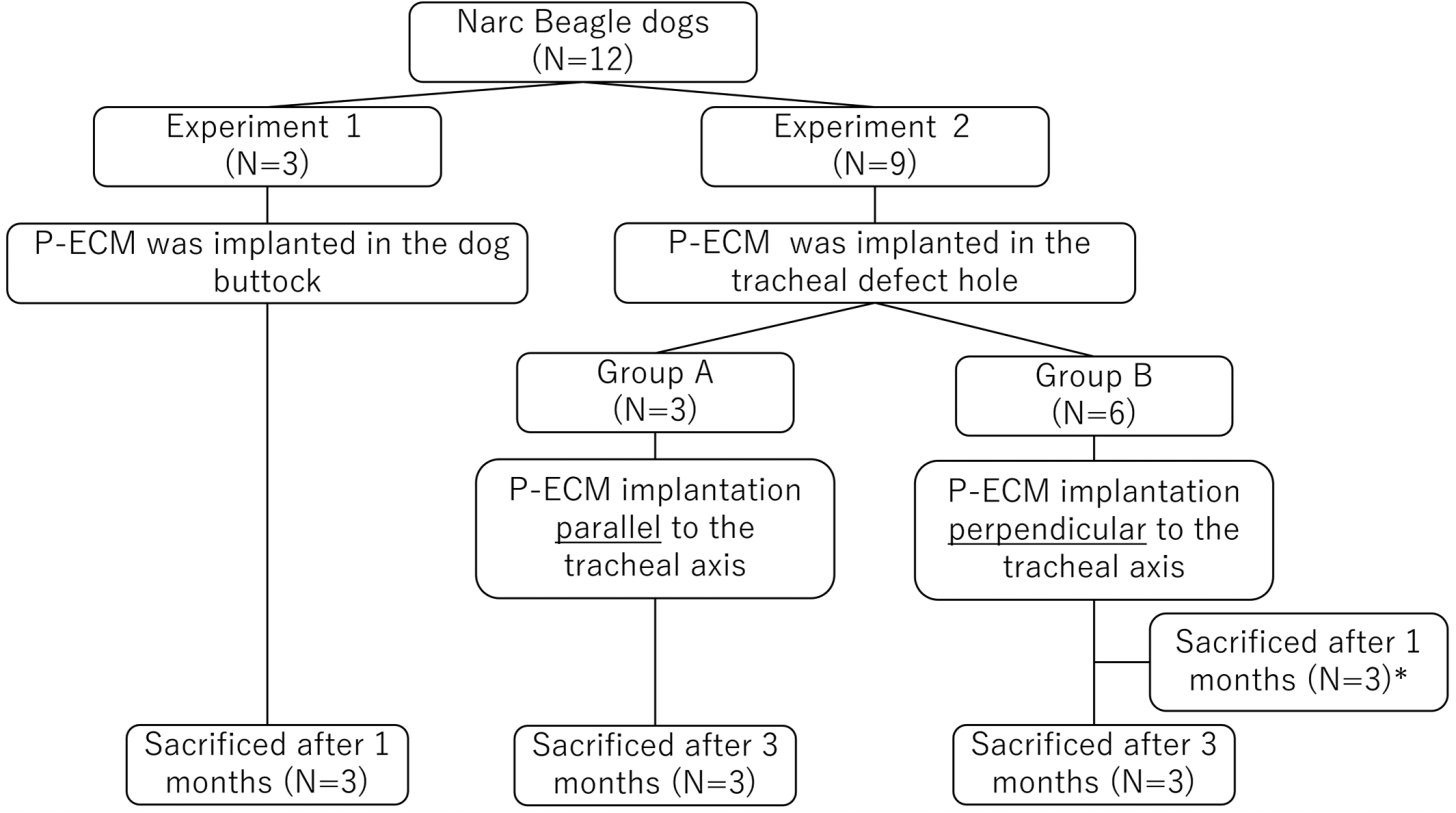
Study design flowchart. *In group B, three dogs were euthanized 1 month after surgery to observe the extent to which epithelialization had progressed.

**Figure 2:**
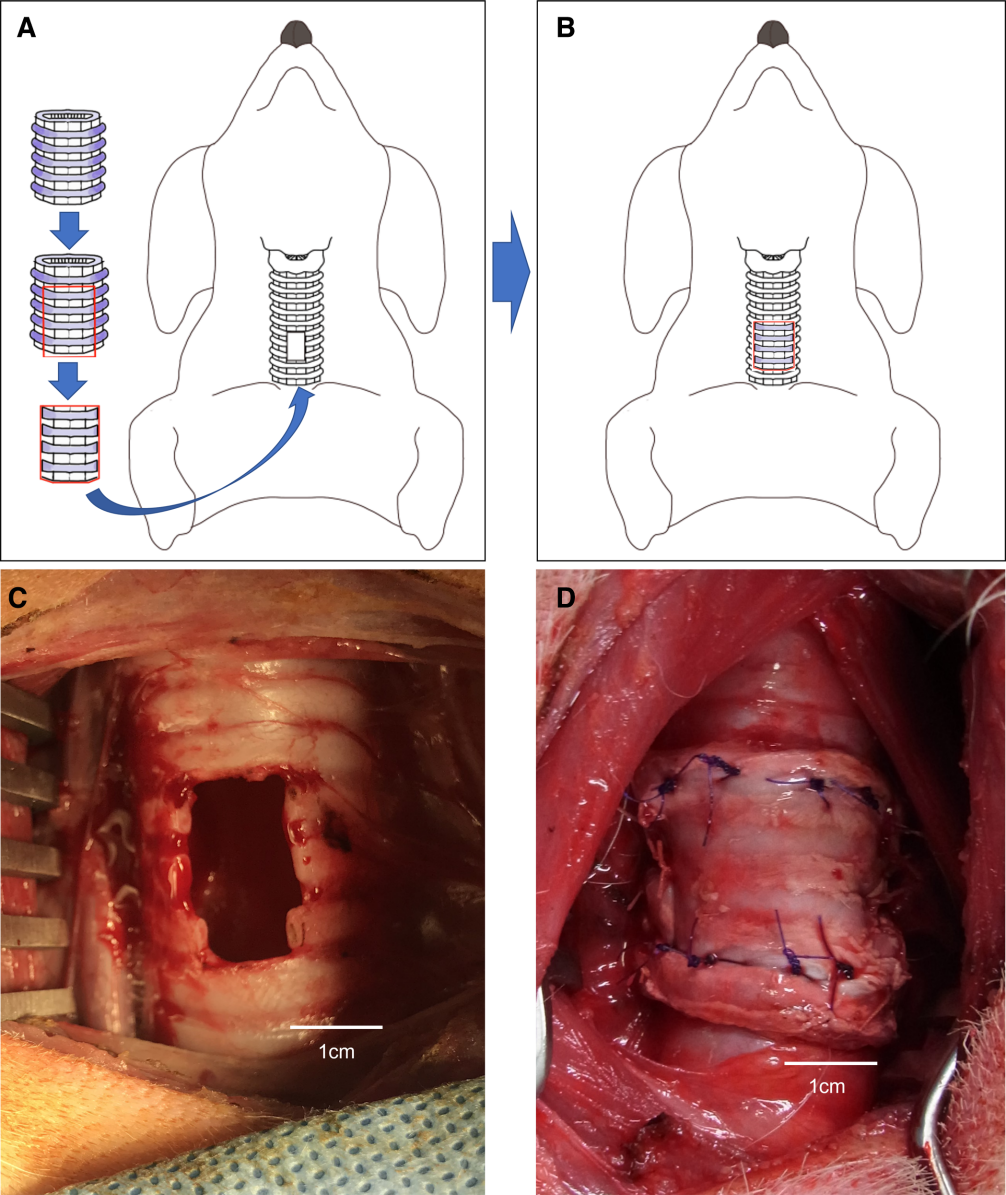
(**A,B**) Schema of the porcine extracellular matrix (P-ECM) transplanted parallel to a tracheal defect (hole) in a dog. (**C**) Tracheal defect (hole) in a dog. (**D**) Porcine extracellular matrix (P-ECM) transplanted parallel to the tracheal defect (hole) in a dog.

**Figure 3:**
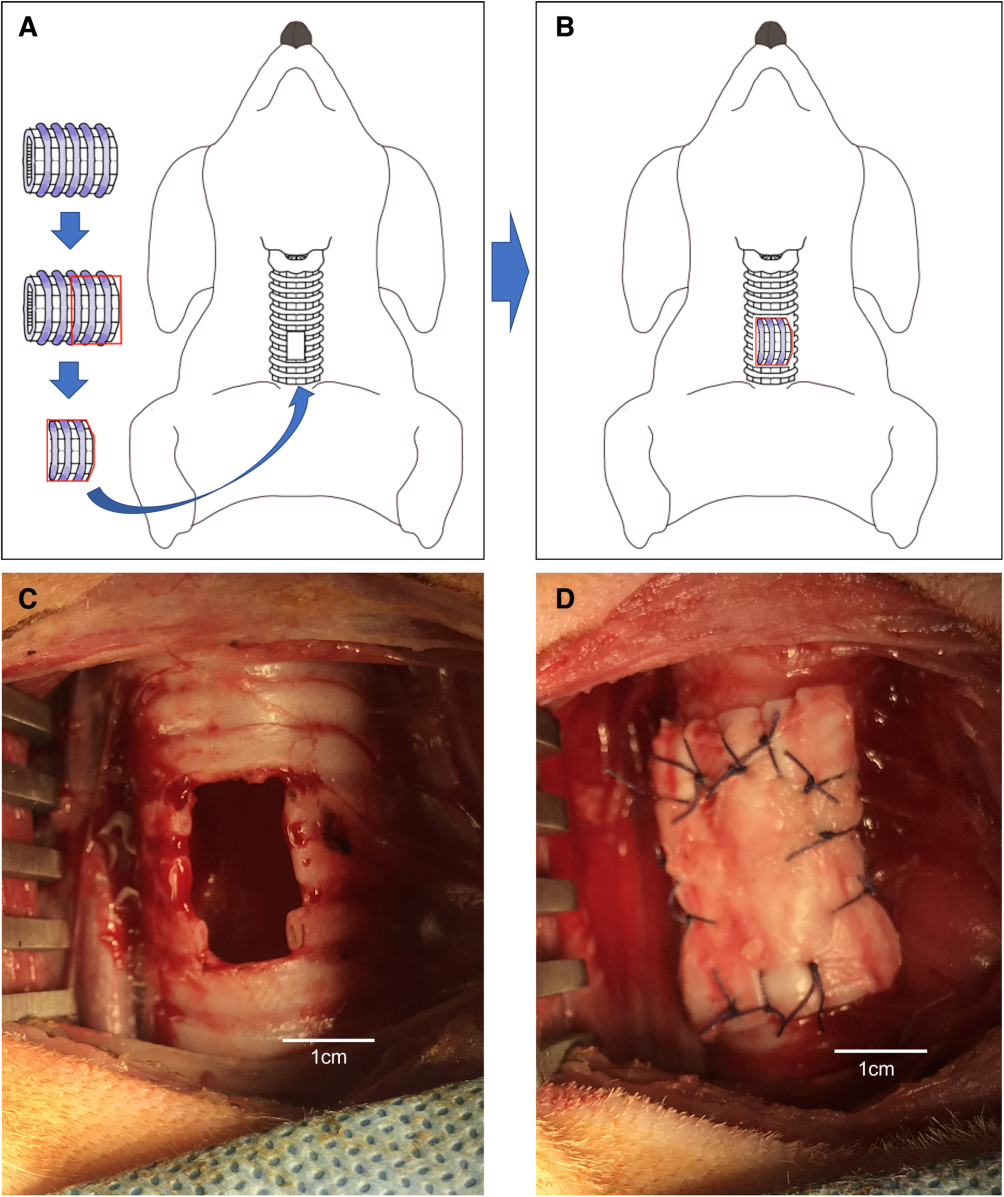
(**A,B**) Schematic of the porcine extracellular matrix transplanted perpendicular to a tracheal defect (hole) in a dog. (**C**) Tracheal defect (hole) in a dog. (**D**) P-ECM transplanted perpendicular to a tracheal defect (hole) in a dog.

A diagram showing the overall study design is shown in Figure 1. First, P-ECM was implanted in dog buttocks (*n* = 3) to observe host responses to P-ECM, including immunologic rejection. The three dogs that underwent surgery were euthanized 1 month after surgery. After confirming that the P-ECM elicited no rejection, orthotopic transplantation of P-ECM was performed. Nine dogs underwent surgical resection of a 1 × 2 cm defect (approximately 30% of the circumference and three rings long) of the ventral cervical trachea. Based on previous study (9), we were concerned about the rigidity of the P-ECM in response to changes in the airway pressure when the matrix was implanted parallel to the tracheal axis. Therefore, in the present study, P-ECM implantation was performed using two different methods: in group A the P-ECM was implanted parallel to the tracheal axis (*n *= 3) (Figures 2A,B), whereas in group B the P-ECM was implanted perpendicular to the tracheal axis to ensure rigidity (*n *= 6) (Figures 3A,B). By doing so, we hypothesized that the cartilage component of the P-ECM could maintain rigidity by vertically bridging the defect hole. The grafts in both groups were observed periodically by bronchoscopy. The animals in group A were euthanized 3 months after surgery. In group B, three dogs were euthanized 1 month after surgery to observe the extent to which epithelialization had progressed and the remaining three dogs were euthanized 3 months after surgery. Morphological changes in the excised P-ECM were evaluated macroscopically and microscopically. The study was performed in accordance with the Guide for the Care and Use of Laboratory Animals prepared by the Institute of Laboratory Resources at the National Research Council (http://nap.edu/catalog/12910.html) and followed the rules for animal experiments of Kagawa University following the approval of the study design by the Committee for Animal Experiments at Kagawa University (authorization number: 21,684).

### 2.2. P-ECM preparation and storage

**Figure 4:**
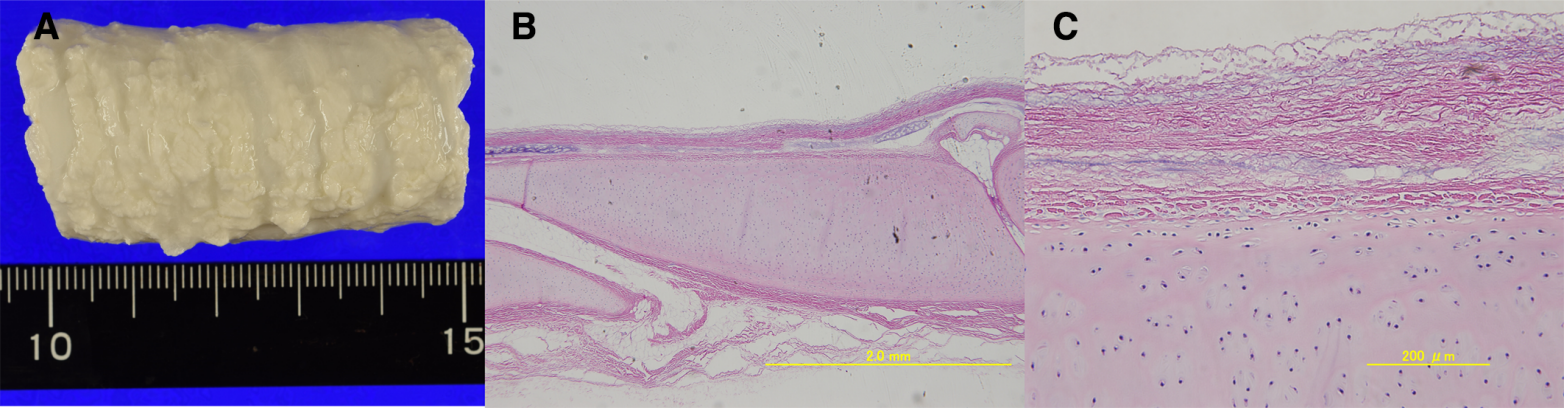
(**A**) Macroscopic view of the extracellular matrix (ECM) created using 17 detergent-enzymatic method cycles. (**B**) Microscopic view (4×) of hematoxylin and eosin histologic transversal sections. at first glance, decellularized porcine trachea appears similar to native trachea. (**C**) Microscopic view (20×) shows removal of tracheal epithelial cells and glands in the ECM. Furthermore, the nuclei of the chondrocytes remained within the cartilage rings, but their cell walls had disappeared.

Porcine tracheae were stored at 4 °C after disassembly and arrived after 24 h of storage. On arrival, the tracheae were immediately washed with purified water and cut to a length of approximately 5 cm. The decellularization process was conducted as previously described (8). Briefly, cut porcine tracheae were treated with 4% sodium deoxycholate (Sigma-Aldrich, St. Louis, MO, USA) for 4 h and perfused with DNAse (1 M NaCl, 2,000 KU DNase; Sigma-Aldrich) for 3 h according to the detergent-enzymatic method, followed by 1 day of storage in purified water. Two days was counted as one cycle of decellularization. Decellularized P-ECM was prepared using 17 such detergent-enzymatic processing cycles (Figure 4). The prepared P-ECM was immersed in phosphate-buffered saline containing 1% antibiotic (penicillin-streptomycin) and antifungal solution (amphotericin A) (both from Sigma-Aldrich), and was stored at 4 °C until use. The non-antigenicity of this matrix has been previously verified; moreover, its physical properties and strength are comparable to those of the native porcine trachea (9).

### 2.3. Surgical procedure

The same anesthetic technique was used in all surgeries. Each dog was anesthetized with ketamine (10 mg/kg), xylazine (5 mg/kg), and atropine sulfate (0.05 mg/kg) administered intramuscularly in the neck. Anesthesia was maintained with continuous infusion of propofol at a rate of 20 mg/kg/h. Each dog was intubated with an 8 mm endotracheal tube and artificial respiration was performed. The ventilator was set to volume control mode and delivered a tidal volume of 20 ml/kg at 15 breaths/min with 100% pure oxygen (inspiratory oxygen fraction = 1.0), inspiratory/expiratory ratio of 1:1, and end-expiratory pressure of 5 cmH_2_O. A pulse oximeter was attached to the tongue. General conditions were monitored using a BSM-3592 instrument (Nihon Kohden Corp., Tokyo, Japan).

### 2.4. Implantation of P-ECM in the dog buttock

Three dogs underwent the P-ECM implant surgery. The dogs were placed in the prone position and an incision was made in the right buttock to dissect the subcutaneous tissue and expose the gluteus medius. A space was created between the muscle layer and subcutaneous tissue of the buttock, and the tubular P-ECM (2.5 cm in length) was placed in the created space. The skin was sutured in the usual manner. After 1 month, the implantation site was inspected and the graft was removed for microscopic assessment.

### 2.5. P-ECM implantation into canine tracheal defects

#### 2.5.1. P-ECM implantation parallel to the tracheal axis (group A)

Orthotopic transplantation of the P-ECM was performed under general anesthesia in three Narc Beagle dogs. Each dog was placed in the supine position on the operating table and 1% xylocaine was injected into the wound. Tracheae were exposed through an anterior midline cervical incision. A tracheal defect (4 ring of cartilage, 1 × 2 cm long) was created on the anterior surface of the trachea, starting 4 cm caudal to the cricothyroid cartilage (Figure 2C). Patchy trimmed P-ECM (2.0 × 3.0 cm) was placed parallel to the defect (Figure 2D). Stitches were placed on each side (cephalad, caudal, right side, left side) with 4–0 absorbable polygalactin (Vicryl; Ethicon, Somerville, NJ, USA) using interrupted sutures. The skin was sutured in the usual manner after confirming airtightness to an airway pressure of 25 cmH_2_O.

#### 2.5.2. P-ECM implantation perpendicular to the tracheal axis (group B)

Perpendicular implantation of the P-ECM was performed under general anesthesia in six Narc Beagle dogs. A tracheal defect was created in the trachea in the same manner as in group A (Figure 3C). The patchy trimmed P-ECM (2.0 × 3.0 cm) was placed in the tracheal defect (hole) so that the P-ECM was perpendicular to the trachea (Figure 3D). After implantation of the P-ECM, the surgical procedure was performed in the same manner as that described for group A.

### 2.6. Postoperative care

All dogs received cefmetazole (0.5 g) (Alfresa Pharma Corp., Osaka, Japan) intramuscularly in the neck from the surgery day until postoperative day 2. The dogs recovered from anesthesia, were extubated and monitored until they rested comfortably in a sternal position. The day after surgery (second day), oral ibuprofen (10 mg/kg) was administered for analgesia. No immunosuppressive medication was administered to the dogs after surgery.

### 2.7. Bronchoscopic assessment

The transplantation site was observed postoperatively, and at postoperative day 3 and weekly thereafter until euthanasia by bronchoscopy. Each time a bronchoscopy was performed, the dogs were anesthetized with ketamine (10 mg/kg), xylazine (5 mg/kg), and atropine sulfate (0.05 mg/kg) administrated intramuscularly in the neck. The degree of tracheal stenosis was visually quantified as the percentage decrease in the ventral dorsal diameter of the trachea. Stenosis was categorized as non-stenosis (0%), mild (25%), moderate (25%–50%), or severe (50%). Recorded bronchoscopic data included the appearance of the graft surface and its relationship to the host trachea (ECM live-implantation rate), signs of inflammation (e.g., granulation tissue), presence of tracheomalacia, and stenosis formation.

### 2.8. Morphological assessment

All dogs were anesthetized (10 mg/kg ketamine, 5 mg/kg xylazine, and 0.05 mg/kg atropine sulfate administrated intramuscularly) and euthanized by intravenous administration of 100–200 mg/kg sodium pentobarbital. The graft site and surrounding tissue (including native tracheal tissue) were harvested immediately after euthanasia. The excised samples were incised in the membranous part, parallel to the tracheal axis, to expose the mucosal surface. The samples were then immersed in 10% neutral buffered formalin for histological preparation. The degree of longitudinal shrinkage of the graft site at 3 months postoperatively was evaluated. The tracheal defect was created with a length of 4 cartilage rings, and was evaluated based on how many cartilage rings remained macroscopically visible at the graft site. For example, if 4 cartilage rings were identified at the implantation site, the shrinkage rate would be 0%; if 2 cartilage rings were identified, the shrinkage rate would be 50%; the average contraction of G-A and G-B was statistically evaluated by comparing the mean values. The tissue was trimmed longitudinally, sectioned, and stained with hematoxylin and eosin. The sites examined were the epithelium on the graft, epithelium at the interface with the host tissue, and center of the graft. The degree of epithelialization on the graft was categorized as non-epithelialization (0%), sporadic (25%), moderate (50%), or full covered (100%). Immunostaining was performed to evaluate for rejection and assess epithelialization on the grafts. In Experiment 1, CD3 immunostaining (Leica Biosystems, Wetzlar, Germany) was performed to evaluate for the presence of rejection. In Experiment 2, cytokeratin AE1/AE3 immunostaining (Agilent Technologies, Santa Clara, CA, USA) was performed to confirm epithelialization. Additionally, we evaluated the epithelium using scanning electron microscopy (SEM) (S-900; Hitachi, Tokyo, Japan) to confirm the presence of pseudostratified ciliated epithelium. The epithelialized site on P-ECM was cut into 3 × 5 mm pieces, which were fixed with 2.5% glutaraldehyde and post-fixed with 1% osmium tetroxide. The samples were then stained with tannin-osmium conductive staining. The samples were dehydrated in a graded ethanol series, immersed in isoamyl acetate, and dried using the critical point drying method (11). Finally, the samples were coated with osmium and observed through SEM.

### 2.9. Statistical analyses

Bronchoscopic stenosis rate, graft epithelialization rate, shrinkage rate and ECM live-implantation rate between the two groups were compared using Mann–Whitney *U* test. A *p*-value of 0.05 or less were considered statistically significant. Statistical analyses were performed with EZR (Saitama Medical Center, Jichi Medical University, Saitama, Japan), which is a graphical user interface for R (The R Foundation for Statistical Computing, Vienna, Austria) (12). More precisely, it is a modified version of R commander designed to add statistical functions frequently used in biostatistics.

## 3. Results

### 3.1. Assessment of P-ECM after decellularization

Seventeen cycles of detergent–enzymatic treatment were sufficient to decellularize the P-ECM of tracheal epithelial cells and glands; nonetheless, nuclei were still visible inside the cartilage ring, whereas cell walls disappeared (Figure 4).

### 3.2. Postoperative course

All dogs underwent transplant surgery and no complications occurred during the procedure. Postoperative complications included suture failure in one dog of group B: marked subcutaneous emphysema appeared at postoperative day 7, after which the dog was immediately anesthetized and emergency reoperation was performed. The defective sutures were re-sutured, and the dog was euthanized 3 months later as planned without any additional complications. None of the other dogs showed any obvious complications until they were euthanized.

#### 3.2.1. P-ECM implantation in the dog buttock

In the postoperative course, all dogs were in the same condition as before surgery from postoperative day 1. The dogs showed vigor and no obvious deterioration in their physical condition, such as decreased food intake. Macroscopically, the matrix was present in the implantation site and no signs of rejection or infection (determined by edema) were confirmed.

**Figure 5:**
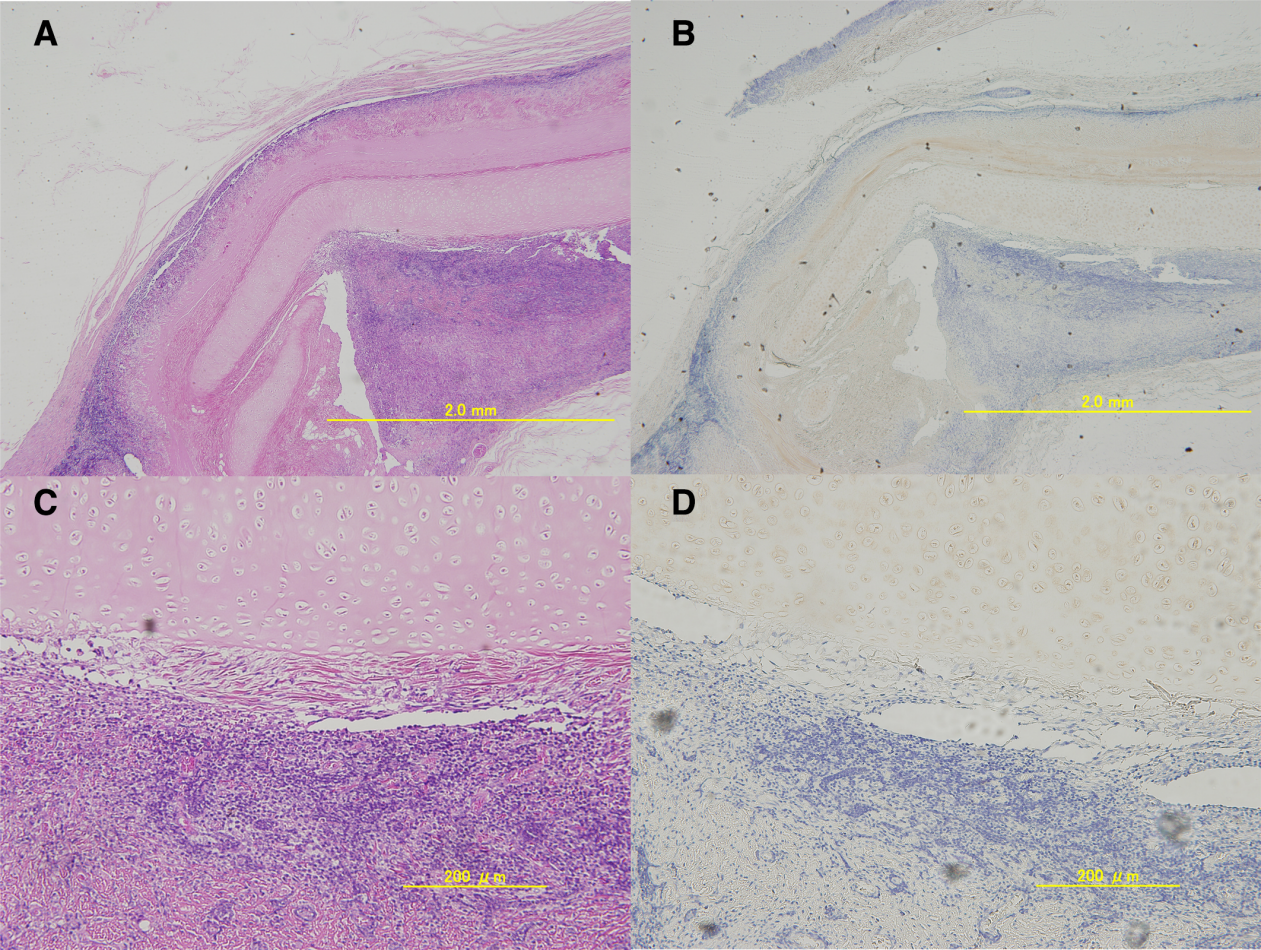
Microscopic images of the excised porcine extracellular matrix (P-ECM) ((**A**); 4×, (**B**); 20×).longitudinal hematoxylin and eosin-stained tissue section. An infiltrate of lymphocytes and polynuclear leukocytes was observed around the decellularized cartilage tissue. However, CD3 staining((**C**); 4×, (**D**);20×) was clearly negative and no obvious rejection was observed.

The longitudinal section of the HE-stained tissues showed that the matrix maintained its morphology. Inflammatory cell infiltration around the matrix was observed (Figures 5A,C). However, no melting or necrosis of the matrix was observed and CD3 staining was negative, confirming that the matrix can exist *in vivo* in a heterologous host without apparent rejection (Figures 5B,D).

#### 3.2.2. P-ECM implantation into canine cervical tracheal defects

##### 3.2.2.1. Bronchoscopic assessment

**Figure 6:**
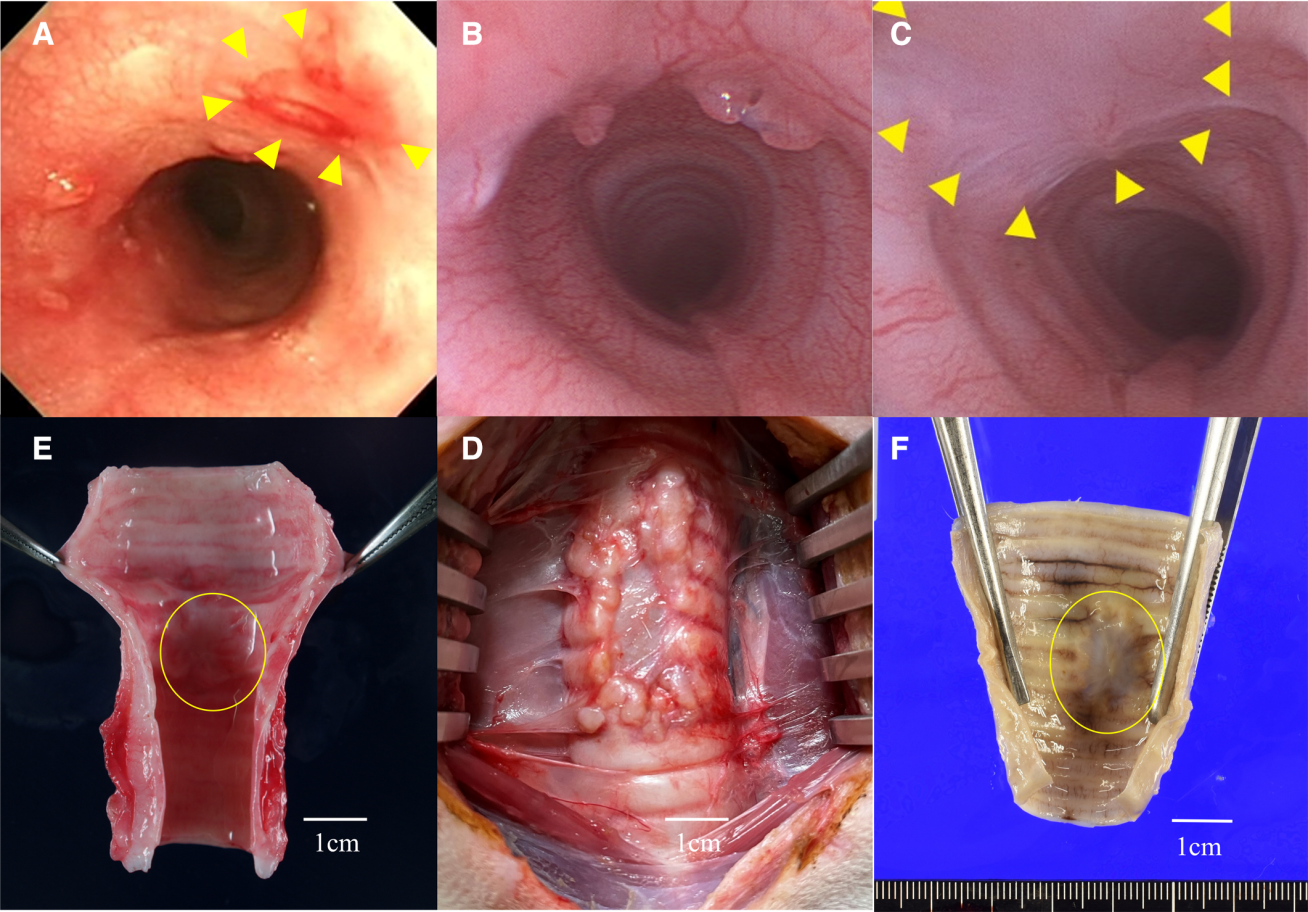
(**A**) Group **A**: bronchoscopic view of the graft site at3 month after surgery. The graft site showed mild redness and scarring, and the lumen was mildly constricted. (**B**) Group **B**: bronchoscopic view of the graft site at 1 month after surgery. Granulation around the graft was noted and the lumen was mildly narrowed. (**C**) Group **B**: bronchoscopic view of the graft site at 3 months after surgery. Granulation was resolved and no stenosis was observed. It was difficult to distinguish the border between the graft and host sites, and the epithelium was continuous. Triangles indicate the grafted area. (**D**) The circled area indicates the porcine extracellular matrix (P-ECM). In group **A** (G-A), the P-ECM site was shrunken, and the original defect site showed longitudinal contraction. (**E**) The P-ECM at three months after surgery in group **B** (G-B) the graft site was replaced with a thin membrane tissue. (**F**) In G-B, contractile changes around the P-ECM were not noticeable.

At postoperative week 1, the edematous changes around the graft site were strong and difficult to evaluate in both study groups. Two weeks after surgery, the edematous changes disappeared and the graft site became easy to observe. One month postoperatively, mild stenosis was observed around the graft site (Figure 6B). Although mild stenosis was observed in both groups, the tracheal lumen was narrower in group A as compared with that in group B. Granulation was observed in the host epithelium near the graft site.

The bronchoscopic findings differed between the two groups. In group A, no improvement in stenosis was observed until 3 months after surgery. Epithelialization of the graft site was not evident, and the ECM site appeared scarred and faded (Figure 6A). In contrast, in group B, the stenosis gradually improved at 2 and 3 months after surgery. Indeed, no stenosis was observed at 3 months and bronchoscopic imaging showed that the boundary between the P-ECM and host site was indistinct with continuous epithelium (Figure 6C). At the P-ECM site of group B, neovascularization was observed beyond the graft site.

##### 3.2.2.2. Macroscopic assessment

There was little adhesion between the P-ECM and the surrounding tissue, and the P-ECM was easily removed. Gross findings at the time of extraction showed that the cartilage component derived from the P-ECM had disappeared in both groups. In group A, the P-ECM site had shrunken and the host site appeared to compensate for the defect by longitudinal contraction (Figure 6D). In group B, 1 month after surgery, the P-ECM site had a very soft structure and the boundary with the surrounding tissue was indistinct. Notably, no obvious narrowing of the bronchial lumen was observed at 3 months and the boundary with the surrounding muscle layer was relatively clear. New blood vessels were found within the graft, which were slightly more visible at 3 months than at 1 month. White structures were observed in the grafts (Figure 6E). The lumen was shiny, suggesting epithelial covering at the graft site (Figure 6F).

##### 3.2.2.3. Statistical assessment

Table 1 showed the details. Bronchoscopic stenosis rate was assessed (G-A; mild (25%) 1/moderate (25%–50%) 1/severe (50%) 1, vs. G-B; non-stenosis (0%) 3). The *p*-value was 0.063, with no significant difference between the two groups. Graft epithelialization rate was assessed (G-A; sporadic (25%) 3, vs. G-B; full covered (100%) 3). The *p*-value was 0.047, indicating a significant difference between the two groups. Shrinkage of the implanted area was assessed. In G-A, there was strong longitudinal shrinkage, and in all three cases only two rings of cartilage were visible in the grafted area (mean shrinkage rate: 50%). In contrast, G-B showed almost no shrinkage: 3 rings of cartilage were visible in one case, and 4 rings of cartilage were visible in the other two cases as before transplantation (mean shrinkage rate: 8.33%, range 0%–25%). Means shrinkage rate between the two groups were compared by Mann–Whitney *U* test, with no significant difference between the two groups. (*p* value;0.059). ECM live-implantation rate was 100% in all 6 cases regardless of G-A or G-B, with no significant difference.

**Table 1:** Comparison of stenosis rates, etc. between parallel and perpendicular group. There was a significant difference between the two groups in graft epithelialization rate. There were no significant differences in other parameters such as shrinkage rate between the two groups.

	Group A (*N* = 3)	Group B (*N* = 3)	*p* value
**Bronchoscopic stenosis rate**			
Non-stenosis (0%)	0	3	
Mild (25%)	1	0	0.063
Moderate (25–50%)	1	0	
Severe (50%)	1	0	
**Graft epithelialization rate**			
Non-epithelialization (0%)	0	0	
Sporadic (25%)	3	0	0.047
Moderate (50%)	0	0	
Full covered (100%)	0	3	
Shrinkage rate (mean)	50/50/50 (50.0)	0/0/25 (8.33)	0.059
ECM live-implantation rate (mean)	100/100/100 (100)	100/100/100 (100)	NaN

##### 3.2.2.4. Morphological assessment

**Figure 7:**
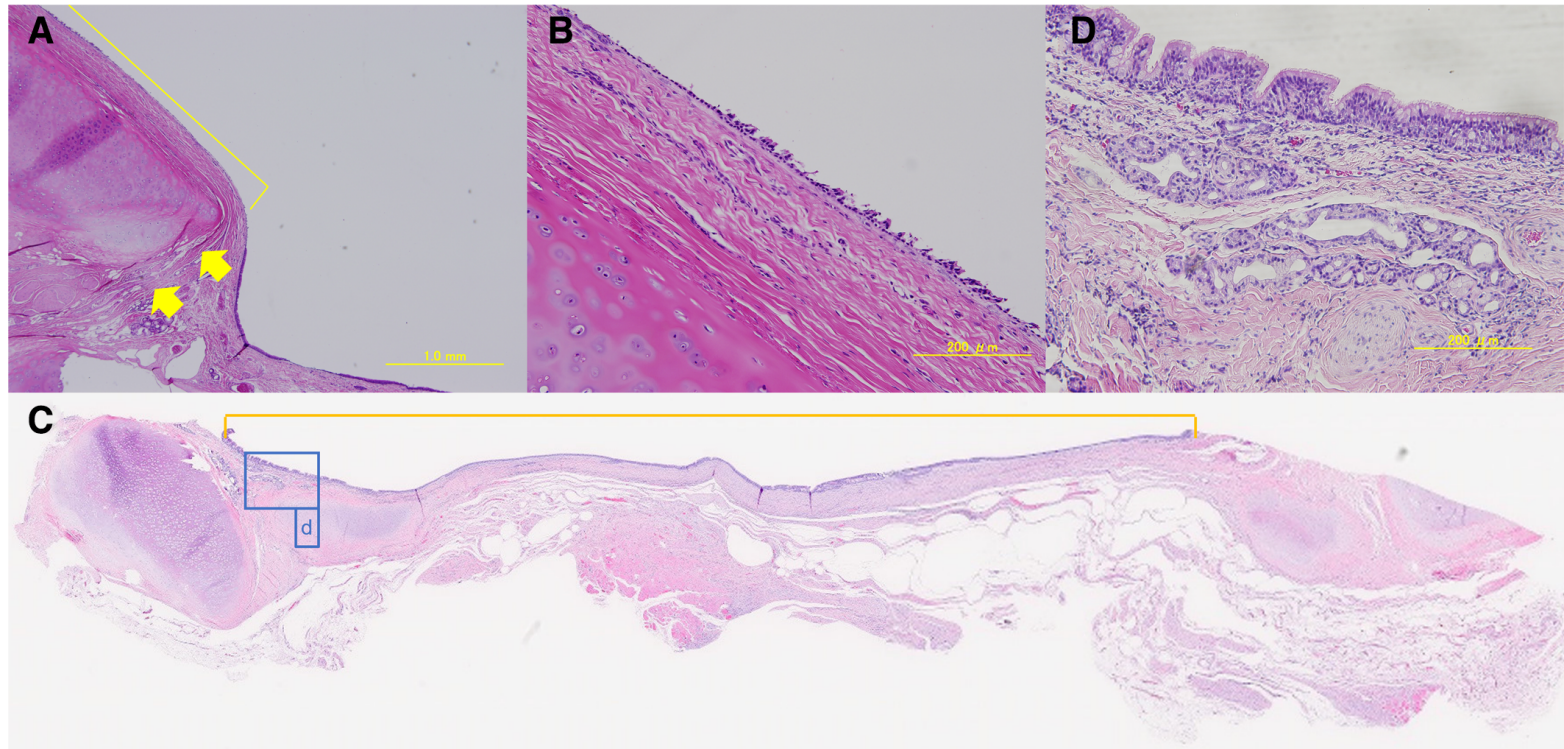
(**A,B**) Group **A**: hematoxylin-eosin-stained sample of the graft-host boundary (4× and 20×). Boundaries are indicated by arrows. The yellow line indicates the graft site. Epithelialization was poor. (**C**) Group **B**: hematoxylin-eosin-stained sample of the graft-host boundary (loupe). The inside of the scale is on the graft site. The tracheal epithelium was present in a continuous border plane. The area surrounded by a blue line (**D**) indicates the graft site. (**D**) Group **B**: hematoxylin-eosin-stained graft sample (20×). Pseudostratified ciliated epithelium, bronchial glands, and elongation of blood vessels, which are structures of normal tracheal epithelium, were observed.

Although no postoperative immunosuppressive drugs were administered, no obvious rejection was observed in either of the groups. In all cases, no chondrocyte generation was observed in the P-ECM. Hematoxylin-eosin staining of group A tissue samples showed no infiltration of inflammatory cells into the graft site, which was reduced and partially replaced by thin connective tissue. Epithelialization of the graft site was poor and only partially observable (Figures 7A,B). In contrast, the cartilage frame had disappeared in group B and inflammatory cells were observed 1 month after surgery (data not shown). At 3 months, no reduction in the grafted area was observed in group B, unlike in group A, and the inflammatory cell infiltration seen at 1 month was no longer observed. In the tracheal lumen, the epithelium was continuous from the host to the graft site and epithelial regeneration was observed (Figure 7C,D).

**Figure 8:**
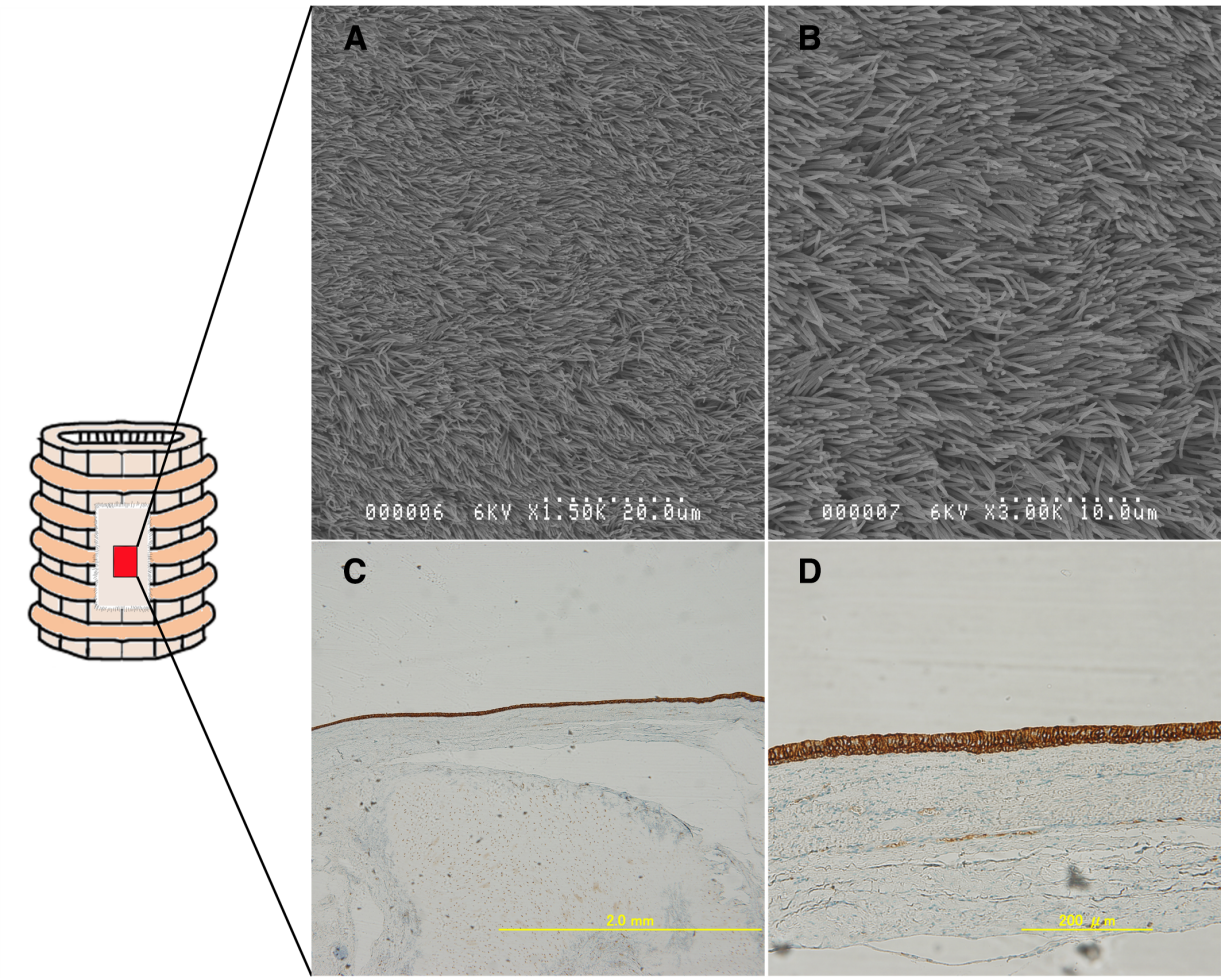
Epithelialization on the implanted ECM was assessed (within the red area). Group **B**: Cytokeratin AE1/AE3-stained samples((**A**); 4×, (**B**); 20×). The staining pattern was consistent with the presence of healthy epithelium. (**C,D**) Scanning electron microscopy images showing the regenerated pseudostratified, densely populated ciliated epithelium. The epithelium is indeed regenerated on the ECM.

The epithelium on the graft clearly showed a pseudostratified ciliated epithelium, bronchial glands, and vascular growth. It has been suggested that regeneration of the tracheal epithelium occurs by perpendicular grafting. SEM analysis confirmed the dense presence of a pseudostratified ciliated epithelium in the tissue sections of group B (Figures 8A,B). In CKAE1/AE3 staining, epithelial cells were stained, confirming the presence of epithelium (Figures 8C,D). In group A, hematoxylin-eosin staining showed little epithelialization, thus immunostaining and SEM were not performed.

In Table 1, the results of morphological and histological assessment of the P-ECM are summarized.

## 4. Discussion

Surgery is the first choice for the treatment of tracheal malignancies, if resectable. The maximum distance that can be resected and reconstructed is approximately 5–6 cm (1, 9). If further resection is necessary, medical materials are required to fill the defect. However, tracheal replacement is not always a life-saving procedure compared to other vital organs, such as the liver, kidneys, and heart (4). Rather, what is desired in daily clinical practice is the development of repair methods for airway defects encountered in perioperative complicated situations (13).

Several synthetic materials have been reported to have the ability to satisfy the mechanical and functional requirements of the trachea (14, 15). However, synthetic scaffolds always harbor the concerns of compliance mismatch with native tissues, which can result in graft stenosis and failure (15, 16). Furthermore, seeding mesenchymal stem cells and/or other recipient cells on the surface of scaffolds may promote tracheal graft survival; nonetheless, the cell seeding process holds additional challenges, such as infection or bacterial contamination (5, 17). Some tracheal defect may be repaired by applying the adequate material without requiring cell seeding process, but only if the graft is epithelialized after being incorporated and engrafted in the host tissue (13, 18). Therefore, a biomaterial that can support the repair of airway defects without requiring cell seeding may contribute to saving patients in life-threatening situations, such as bronchopleural fistula or empyema (19).

We previously reported a case in which the bronchial wall of a resected lung was trimmed and used as a bronchial patch for autotransplantation (13). However, it is not possible to harvest bronchial patches from a patient and autotransplantation cannot always be performed. Although various studies have explored the potential of ECM as a xenogeneic material (10), there are few reports on their use in large animals. Given that this ECM is nonantigenic and is not rejected after transplantation on the back of mice (9), we aimed to explore its potential application as an airway repair and xenogeneic biomaterial. As repair material for airway surgery, the P-ECM has unique features, including its airtightness, ease of suturing to the airway wall, and no need for immunosuppression despite its xenogeneic nature. Moreover, xenogeneic scaffolds from porcine trachea have an advantage in terms of supply, as porcine tissues are commercially available.

We had two concerns in this study. One was the host response to P-ECM as a xenogeneic material. We used a detergent-enzymatic method that was unable to fully remove all nuclei of chondrocytes; however, chondrocyte antigens are expressed only on their membrane and not on their nuclei (9). Moreover, it is believed that decellularized ECM does not have a vascular structure; thus, necrosis due to rejection is unlikely to occur (20, 21). Although ECM derived from porcine tissues contains a small amount of galactose-α-1,3-galactose epitope, it is believed that such a small amount is not sufficient to induce complement activation as a rejection reaction (20, 22). Considering these, we conducted the heterotopic transplantation of the P-ECM in the right buttock of dogs. We confirmed that no rejection reaction, such as edema or abscess formation at the transplanted site, occurred. The dogs were always in good condition until euthanasia (data are not shown). As cytotoxic T cells are the main effectors of tissue rejection, we evaluated whether CD3^+^ mononuclear cells infiltrated the tissues surrounding the P-ECM. Microscopic findings showed that the P-ECM was not damaged by CD3^+^ cells and no rejection reaction was induced, although some inflammation was noticeable.

The second concern was the rigidity of the P-ECM (9), which was a disadvantage of decellularization process. Indeed, remnant nuclei in the ECM were suggested to have a positive effect on the preservation of its mechanical characteristics (23). Therefore, in addition to the usual transplantation parallel to the tracheal axis (group A), we also performed patch transplantation perpendicular to the tracheal axis (group B). We expected that the remnants on the cartilage after decellularization would act as a stiffening aid (9). Implantation perpendicular to the tracheal axis might be a concerning point in surgery; however, overlying the P-ECM on the tracheal defect was a simple procedure and required no special ingenuity.

The present study showed, as in Table 1, that the P-ECM can be incorporated into the host tissue without airway stenosis, as confirmed by bronchoscopic observation, thereby promoting the healing of the tracheal defect, with epithelial cells of the host tissue migrating to and covering the surface of the xenogeneic P-ECM scaffold without cell seeding. The reason why the significant deference was not evident on bronchoscopic stenosis rate and shrinkage rate might be simply due to the small number of specimens. Notably, no xenograft rejection was observed, even without immunosuppression. These results demonstrate the clinical potential of P-ECM as a useful material for repairing airway defects.

The details of how the ECM graft reacts with host tissue remain unknown. In our experimental group A, the ECM eventually incorporated and the defect healed but with a stenotic scar. In contrast, the mucosal connective tissue of the ECM grafts in group B was fully epithelialized and the defect was healed without stenosis. The different results between the two groups may be explained by the positive effect of chondrocyte nuclei on the mechanical characteristics of the ECM (23). In group A, the ECM architectural integrity collapsed because the graft was not sufficiently strong to resist the negative pressure of respiration owing to its insufficient rigidity. Consequently, innate growth factors of ECM, such as the basic fibroblast growth factor, cannot be activated and it is possible that the ECM-host response was not successfully induced (20, 24, 25). In situations in which the host-ECM response process does not proceed well, ECM degradation does not occur adequately and epithelialization is not promoted (10, 26). In addition, implanted trachea ECM patches are always exposed to the risk of airway infection, which may lead to scar tissue formation due to chronic inflammation (27). In group B, we speculate that perpendicular implantation of the graft to the host trachea axis can maintain a stronger grip between the defect edges than horizontal implantation. This could secure the time for ECM degradation and initiate the reciprocal host-ECM interaction, which are required for tissue remodeling (25, 28) The ECM architecture, especially the cartilage part, is durable enough to withstand the negative respiratory pressure during the adequate remodeling process in group B because the chondrocyte architecture in the ECM was preserved. After implantation of the graft in our experimental group B, degradation of the ECM, especially the ECM mucosal connective tissue, began. The interaction between the degradation products of the ECM and the innate ECM growth factors may activate migration of the host epithelial cells, thereby resulting in epithelialization of the surface of the ECM scaffold with host cells (24, 25, 29, 30).

Regarding the application of a porcine trachea ECM to dogs as a xenograft material, it could be said that pigs and dogs are not strictly xenogeneic considering their phylogenic proximity. On the other hand, pigs organs have been used as a xenotransplant material for humans, and many experiments and applications have been conducted (31, 32) Although xenotransplantation poses a problem of its antigenicity, decellularization process could eliminate its xenoantigens including alfa- gal epitopes. In addition, in the decellularization model of the lung, alfa- gal epitopes are said to decrease or disappear when the lung ECM is recellularized (33). Moreover, the use of organs from alpha 1,3-galactosyltransferase knockout pigs may aid the decellularization process, resulting in a more complete removal of alpha-gal antigens (34). These evidences may support that this P-ECM might have a possibility as a xenogeneic biomaterial subject to further investigation.

This study has several limitations. First, a small number of animals were evaluated at each time point due to practical considerations, including animal welfare and study costs. Second, the assessment were performed during a relatively short time period (3 months), and there was no long-term evaluation of the trachea or serological changes of the immune system. Nevertheless, no serological problems were described in a similar study on mice (9). Third, further studies are necessary to better understand chondrocyte regeneration and promote chondrogenesis on this matrix to ensure rigidity of the P-ECM. Finally, the time frame needed for preparing the matrix using the detergent-enzymatic method is relatively long (approximately 1 month), but it has been suggested that it could be shortened (26).

## 5. Conclusions

We have shown that xenogeneic porcine ECM graft prepared using the detergent-enzymatic method has the potential to be a biological material for airway defect repair in dogs. Full re-epithelialization from the host tissue on the mucosal connective tissue of the xenogeneic scaffold without immunosuppression was confirmed. Adequate methods are still necessary to promote enhanced regeneration of chondrocytes and cartilage formation in the matrix, and further studies are warranted to explore the potential of this ECM as a xenogeneic biomaterial for clinical use that does not require immunosuppression.

## Data Availability

The original contributions presented in the study are included in the article/Supplementary Material, further inquiries can be directed to the corresponding author/s.
